# The spectrum of pregnancy-induced cardiac remodeling: transitioning from healthy adaptive hypertrophy to peripartum cardiomyopathy

**DOI:** 10.3389/fcell.2026.1878716

**Published:** 2026-07-30

**Authors:** Kelly N. Araujo, Pooja Choubey, Michelle L. Matter

**Affiliations:** The Lundquist Institute for Biomedical Innovation at Harbor-UCLA Medical Center, Torrance, CA, United States

**Keywords:** cardiac signaling, genetic variants, heart failure, hypertrophy, mechanotransduction, peripartum cardiomyopathy

## Abstract

Pregnancy imposes profound hemodynamic, metabolic, and biomechanical stresses on the heart, requiring coordinated structural and molecular adaptations to preserve cardiac function. To compensate, the cardiovascular system undergoes significant reversible adaptations, such as increased blood volume and elevated cardiac output. Such alterations induce physiological left ventricular hypertrophy that is regulated, in part, by mechanotransduction, a process by which cells convert mechanical stimuli such as stretch and pressure into biochemical pathways. Integrin-mediated complexes link cardiomyocytes with the extracellular matrix (ECM), allowing the myocardium to sense increased wall stress/stretch and activate cardioprotective signaling cascades. Together, these pathways preserve cytoskeletal integrity, sustain cardiomyocyte viability, and promote adaptive hypertrophic growth that regresses post-partum as hemodynamic load returns to pre-pregnancy levels. However, within a subset of pregnant women, this healthy adaptive response becomes dysregulated and adaptive hypertrophy transitions into maladaptive, leading to peripartum cardiomyopathy (PPCM) with heart failure (HF). PPCM has a mortality rate between 7% and 15%, establishing it as a significant cause of maternal death globally. Here, we discuss integration of mechanotransduction and cell survival pathways to support cardiac adaptation during pregnancy. Elucidating the role of gene variants and mechanotransduction in the pregnant heart may lead to improved patient outcomes in PPCM and HF.

## Introduction

**FIGURE 1:**
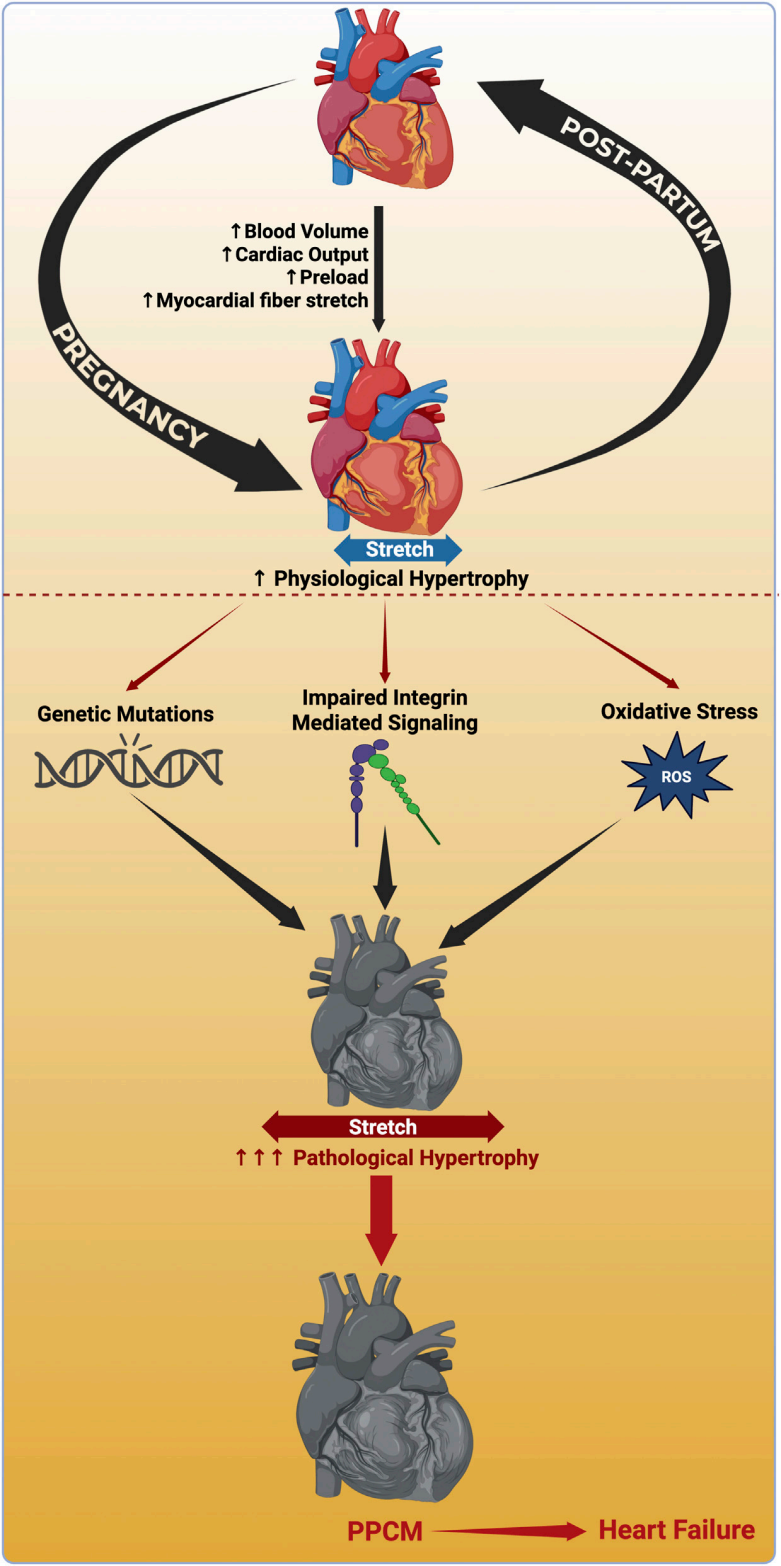
The Pregnant Heart: Progression from physiological to pathological cardiac hypertrophy. (*Top panel*) During pregnancy, increased blood volume induces excessive hemodynamic load on the healthy maternal heart, leading to myocardial stretch (blue double-sided arrow) and initiating physiological cardiac hypertrophy. Under healthy conditions, this adaptive hypertrophy is tightly regulated and preserves cardiomyocytes survival and cardiac function (*Bottom panel*) Dysfunction or loss of regulatory signals, such as genetic mutations, impaired integrin-mediated mechanotransduction or oxidative stress induces sustained, unchecked myocardial stretch (red double-sided arrow) promoting pathological hypertrophy. This maladaptive remodeling impairs cardiac function causing PPCM and subsequent HF. Created in BioRender. Matter, M (2026) https://BioRender.com/14d21ea.

During pregnancy, the heart must adapt to extreme mechanical stress through coordinated alterations in structure and function. The increased hemodynamic load of pregnancy stretches the myocardium, inducing significant but reversible adaptations, including physiological left ventricular hypertrophy (Adeyeye et al., 2016), which is regulated, in part, by mechanotransduction (Garoffolo and Pesce, 2019). Integrin-mediated complexes link cardiomyocytes with the ECM, allowing the myocardium to sense increased wall stress and activate cardioprotective signaling cascades (Laser et al., 2000), such as peptidyl-tRNA transferase (PTRH2) (Montoya-Uribe et al., 2025), phosphoinositol 3-kinase-akt (PI3K-AKT) (Clemente et al., 2012), mitogen-activated protein kinase (MAPK) (Pham et al., 2000; Braz et al., 2002), and signal transducer and activator of transcription 3 (STAT3) (Pan et al., 1999). In addition, metabolic reprogramming, angiogenesis, and ECM turnover are all increased to meet the high demands of pregnancy (Laser et al., 2000; Montoya-Uribe et al., 2025; Clemente et al., 2012; Pham et al., 2000; Braz et al., 2002). Together, these adaptations sustain cardiomyocyte viability and heart function. Indeed, the adaptive hypertrophy that occurs in a healthy pregnancy is tightly regulated and regresses during the postpartum period as hemodynamic load returns to pre-pregnancy levels (Cong et al., 2015; Umar et al., 1985) (Figure 1 *Top Panel*). However, when this healthy adaptive response becomes dysregulated it causes PPCM and subsequent HF. In the absence of coordinated mechanotransduction and intracellular signaling, the heart is unable to activate key protective signals to buffer pregnancy-initiated mechanical stress (Lyon et al., 2015; Chung and Leinwand, 2014). In this instance, cardiac hypertrophy is unchecked leading to dilated cardiomyopathy (DCM) and progressing to HF in late pregnancy or within 5 months postpartum (Lyon et al., 2015; Chung and Leinwand, 2014; Iannaccone et al., 2024; Koziol and Aronow, 2023) (Figure 1 *Bottom Panel*).

Recently, RNA seq and genetic sequencing have provided the first analyses of gene variants in PPCM and identified several structural genes that are mutated in PPCM and DCM including titin (*TTN)* and β-myosin heavy chain *(MYH7)* (Goli et al., 2021; Ware et al., 2016; van Spaendonck-Zwarts et al., 2014). How these mutations alter key molecular pathways that regulate adaptive hypertrophy remains to be fully elucidated. A critical unanswered question is*: What safeguards normal adaptive hypertrophy and prevents its progression to pathological hypertrophy and subsequent HF?* This review explores the molecular mechanisms underlying pregnancy-induced adaptive cardiac hypertrophy highlighting the emerging role of key genes*,* in preserving cardiac adaptation during pregnancy.

## The healthy heart: adaptive physiological hypertrophy induced by pregnancy

**FIGURE 2:**
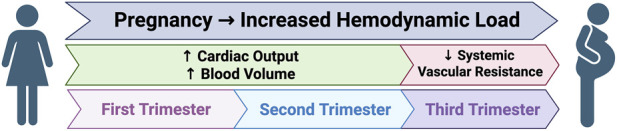
Maternal cardiovascular adaptations during pregnancy. Pregnancy is associated with an increased hemodynamic load characterized by a progressive rise in cardiac output and blood volume, alongside a decrease in systemic vascular resistance. These physiological changes evolve across the three trimesters, supporting the growing metabolic demands of developing fetus. Created in BioRender. Matter, M (2026) https://BioRender.com/orv27kl.

To accommodate the extreme metabolic and circulatory demands of a developing fetus, maternal blood volume significantly increases to 40% total volume during pregnancy, resulting in elevated preload and ventricular filling (Longo, 1983; Ferreira et al., 2024). According to the Frank-Starling law this increased preload stretches myocardial fibers at end-diastole resulting in augmented contractile force and stroke volume (Ferreira et al., 2024; Patterson and Starling, 1914; Patterson et al., 1914). The elevated stroke volume and heart rate significantly increases cardiac output within the first trimester and cardiac output reaches peak levels between 20–30 weeks of gestation, which is sustained during the initial postpartum period (Hunter and Robson, 1992; Robson et al., 1989) (Figure 2). It is this sustained stress that pushes the maternal heart into adaptive cardiac remodeling, characterized by left ventricle hypertrophy (Adeyeye et al., 2016; Umazume et al., 2018; Savu et al., 2012) at 26–30 weeks of pregnancy. Importantly, unlike pathological hypertrophy, adaptive pregnancy-induced cardiac growth preserves systolic function (Umar et al., 1985; Virgen-Ortiz et al., 2009). During the postpartum period, the heart undergoes reverse remodeling, and heart function returns to pre-pregnancy levels (Cong et al., 2015; Umar et al., 1985; Chung and Leinwand, 2014; Sadoshima and Izumo, 1997; Neves et al., 2015; Landim-Vieira et al., 2025; Maillet et al., 2013; Casarella et al., 2024).

**FIGURE 3:**
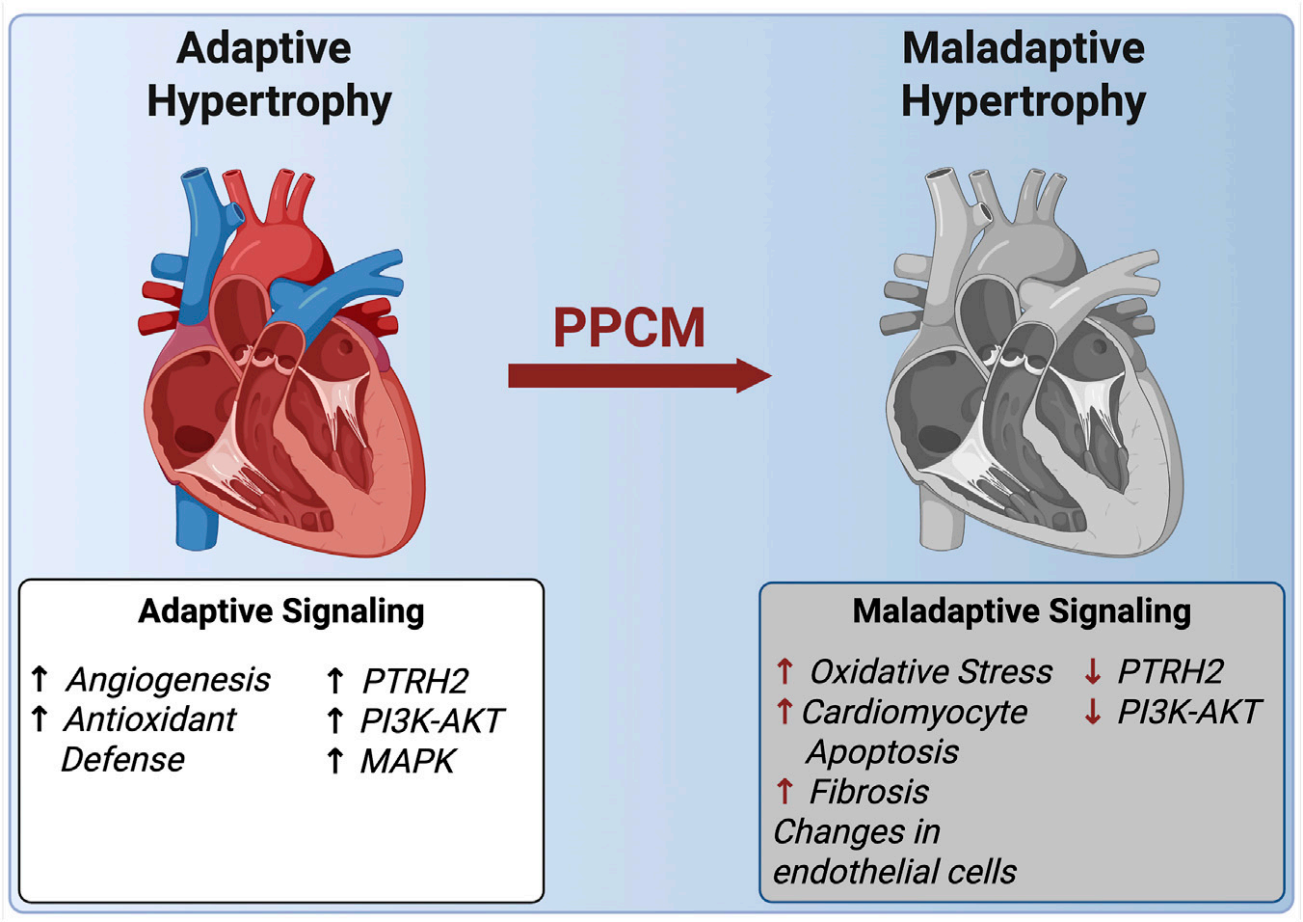
Dysregulation of key signals in the pregnant heart leads to PPCM and HF. Pregnancy increases the hemodynamic load on the maternal heart, typically resulting in adaptive hypertrophy characterized by preserved cardiac function and activation of protective signaling pathways, including increased PI3K-AKT, MAPK, PTRH2. Angiogenesis and anti-oxidant defenses are also upregulated. PPCM shifts responses towards maladaptive hypertrophy, marked by pathological remodeling and impaired cardiac function. This maladaptive state increases oxidative stress and downregulates PTRH2 and PI3K-AKT signaling promoting cardiomyocyte apoptosis, endothelial cell dysregulation, and increased fibrosis inducing PPCM and HF. Created in BioRender. Matter, M (2026) https://BioRender.com/4h4s1yx.

Mechanotransduction pathways enable cells to sense and respond to mechanical cues such as matrix stiffness, stretch, and shear stress (Jalali et al., 2001; Schwartz, 2010). Integrins function as primary mechanotransducers by coupling extracellular mechanical inputs to intracellular signaling cascades to regulate survival, growth, and structural remodeling (Israeli-Rosenberg et al., 2014). In the heart, dysregulation of these pathways disrupts cellular adaptation to mechanical stress and compromises tissue homeostasis leading to pathological hypertrophy and HF (Brancaccio et al., 2006; Harston and Kuppuswamy, 2011; Valencik et al., 2006). Mechanotransduction is regulated, in part, by integrins that are transmembrane heterodimeric receptors composed of α and β subunits. Integrins connect the ECM to the intracellular cytoskeleton. In the myocardium, integrins localize at costameres and intercalated discs where they anchor sarcomeres to the sarcolemma to facilitate mechanical coupling between the adjacent cardiomyocytes (Belkin et al., 1996; Wu et al., 2002). Cardiac integrin heterodimers are α1β1, α5β1, and α7β1, with α7 and β1 subunits being most abundant (Israeli-Rosenberg et al., 2014; Ross et al., 1998; Brancaccio et al., 1998; Okada et al., 2013; Maitra et al., 2000). In response to increased hemodynamic load, integrins sense cardiomyocyte stretch to initiate intracellular signaling through focal adhesion kinase (FAK) (Laser et al., 2000; Lal et al., 2007; Browe and Baumgarten, 2003). Mechanical stretch induces FAK autophosphorylation at Tyr397, allows for recruitment of Src-family kinases and activation of downstream pathways including PTRH2, PI3K-AKT, ERK1/2, and p38 MAPK, all of which regulate hypertrophic growth and cardiomyocyte survival (Laser et al., 2000; Clemente et al., 2012; Pham et al., 2000; Braz et al., 2002; Kuppuswamy et al., 1997; Griffiths et al., 2011) (Figure 3). The integrin-associated scaffolding proteins, talin, vinculin, paxillin, and kindlin, further stabilize focal adhesion complexes and link integrins to the actin cytoskeleton, facilitating efficient transmission of mechanical forces during contraction (Carisey et al., 2013; Humphries et al., 2007; Chen et al., 2019; Li et al., 2025). Sustained or dysregulated mechanotransduction through integrins and second messengers contributes to maladaptive remodeling, fibrosis, and contractile dysfunction, highlighting the delicate balance between adaptive and pathological responses to mechanical stress and strain, contributing to the development of PPCM and HF.

Beyond these canonical pathways, integrin mechanotransduction interfaces with transcriptional and metabolic programs that coordinate cardiomyocyte adaptation to mechanical stress. Emerging evidence suggests that mechanotransduction cues converge on regulators of gene expression and mitochondrial function, linking extracellular forces to energetic remodeling of the heart. *STAT3* is downstream of integrin signaling and links mechanical cues to transcriptional programs to regulate cardiomyocyte survival, hypertrophic growth, and cytoskeletal remodeling (Harhous et al., 2019; Willey et al., 2008; Kunisada et al., 2000; Jacoby et al., 2003). In parallel, *PGC-1α* functions as a regulator of mitochondrial biogenesis and oxidative metabolism, integrating stress-responsive and metabolic signaling pathways (Wu et al., 1999; Cao et al., 2025; Scarpulla, 2011). Integrins can transmit mechanical cues to modulate mitochondrial function and metabolic gene expression and PGC-1α may be a potential mediator of mechano-metabolic coupling in the heart (Cao et al., 2025; Tharp et al., 2021; Liao et al., 2020). However, despite the established roles of STAT3 and PGC-1α in cardiac stress responses, their specific contributions and potential crosstalk with integrin-mediated mechanotransduction pathways within the context of the maternal heart physiology during pregnancy remain to be more fully elucidated. Importantly, metabolic remodeling during pregnancy is not driven solely by increased hemodynamic load and mechanotransduction. Pregnancy is accompanied by profound systemic metabolic adaptations, including alterations in circulating glucose, lipids, ketone bodies, and hormonal signals such as insulin, estrogen, progesterone, and placental hormones (Liu and Arany, 2014; Hadden and McLaughlin, 2009). These changes modify substrate availability and utilization by the maternal heart, promoting a shift in energy metabolism that supports the increased energetic demands of pregnancy. Therefore, cardiac metabolic remodeling likely arises from the integration of both altered substrate availability and mechano-metabolic signaling pathways (Hill, 2019).

Another integrin regulated protein is the peptidyl tRNA hydrolase 2 (PTRH2). It is an evolutionary conserved and ubiquitously expressed protein with diverse roles in cellular homeostasis (Giorgi et al., 2025). While PTRH2 is essential for maintaining efficient translation turnover by hydrolyzing prematurely dissociated peptidyl-tRNA molecules, thereby preventing accumulation of stalled translation intermediates and preserving translational fidelity (Corpuz et al., 2020; De Pereda et al., 2004), it also moonlights to control key cellular functions. Structurally, the PTRH2 protein contains a mitochondrial localization sequence and a catalytic hydrolase domain. Its asymmetric α/β fold, composed of a central β-sheet flanked by two α-helices, suggests a potential role for protein binding at the largest α-helix within the catalytic domain (De Pereda et al., 2004). Beyond its canonical enzymatic function, PTRH2 is a regulator of mitochondrial metabolism (Giorgi et al., 2025), myogenic differentiation (Griffiths et al., 2015), cellular stress responses (Griffiths et al., 2011; Yi et al., 2010; Biliran et al., 2008), and adhesion-dependent survival/cell death signaling (Griffiths et al., 2011; Jan et al., 2004). In the heart, *PTRH2* is an essential mediator of cardiomyocyte mechanical and survival signals (Montoya-Uribe et al., 2025).

Taken together, the sustained mechanical stretch of the maternal myocardium activates mechanotransduction pathways (Elkayam, 2011). These pathways translate mechanical stimuli into intracellular growth and survival signals to initiate balanced hypertrophic remodeling while maintaining cardiomyocyte function (Pearson et al., 2000). In addition to mechanotransduction, emerging evidence suggests that successful maternal cardiac adaptation also relies on (Chung and Leinwand, 2014; Sadoshima and Izumo, 1997; Neves et al., 2015; Landim-Vieira et al., 2025), metabolic remodeling (Sayles et al., 2026), calcium homeostasis (Onusko et al., 2020), and cardiomyocyte stress-survival pathways (Montoya-Uribe et al., 2025; Liu et al., 2018). These interconnected systems translate the sustained mechanical and energetic demands of pregnancy into intracellular signals to support hypertrophic remodeling while preserving cardiomyocyte function and viability.

These tightly regulated processes enable the maternal heart to adapt to the increased hemodynamic load without activating pathological remodeling (Maillet et al., 2013). Identifying the molecular mechanisms that induce physiological hypertrophy under extreme mechanical load will provide potential targeted therapies to block pathological hypertrophy and subsequent HF in PPCM and in other cardiovascular diseases.

## Post-partum cardiomyopathy: transitioning from adaptive to maladaptive hypertrophy

PPCM is a distinct form of HF that develops in late pregnancy or within the first 6 months postpartum without prior structural heart disease or other identifiable causes of HF (Iannaccone et al., 2024). Established risk factors include advanced maternal age, African ancestry, chronic hypertension, preeclampsia, multiple pregnancies, and prolonged administration of pharmacological therapies prescribed to suppress preterm labor (Elkayam, 2011). Diagnosis requires echocardiography evidence of left ventricular systolic dysfunction, defined as a left ventricular ejection fraction <45%, fractional shortening <30%, or both, independent of left ventricular end-diastolic dimension >2.7 cm/m^2^ (Pearson et al., 2000). CVD affects over 4% of pregnancies causing over one-third of United States of America maternal deaths. PPCM alone is responsible for approximately 8%–12% of maternal deaths in the United States of America (approximately one in 1,000 to one in 4,000 live births) (Isogai and Kamiya, 2019; Kolte et al., 2014; Davis et al., 2020; Pathak et al., 2025; Chen et al., 2025). Worldwide, the incidence of PPCM is variable with the highest occurrence in Nigeria and Haiti at 1:100 and 1:300 live births, respectively (Isezuo and Abubakar, 2007; Fett et al., 2005). Japan presents with the lowest incidence at 1:20,000 live births (Kamiya et al., 2011). Overall, PPCM is a major cause of pregnancy-associated cardiovascular mortality and contributes significantly to maternal morbidity and mortality during late pregnancy and the postpartum period (Pathak et al., 2025; Sliwa et al., 2010a).

Genetic mutations are increasingly recognized as underlying causes of PPCM, with pathogenic variants identified in a subset of PPCM patients, particularly in sarcomere and cytoskeletal genes (Table 1). (Goli et al., 2021; van Spaendonck-Zwarts et al., 2014) In a cohort of 172 women with PPCM, rare truncating variants in cardiomyopathy-associated genes, including *TTN* (titin), were identified in approximately 15% of patients, with a mutation burden comparable to those observed in non-ischemic DCM cohorts, indicating a shared genetic susceptibility (Ware et al., 2016; Lim, 2016). Other genes identified in both PPCM and DCM include *DSP* (desmoplakin), *FLNC* (filamin C), *BAG3* (Bcl2-associated athanogene 3), *MYH6* (α-myosin heavy chain), *MYH7* (β-myosin heavy chain), and *LMNA* (lamin A/C) (Goli et al., 2021; Spracklen et al., 2021; Seidman et al., 2025; Morales et al., 2010). Larger next-generation sequencing studies are being performed to clarify the genetic architecture of PPCM. While the genetic information is currently limited in PPCM this cohort is continuing to increase. For example, 469PPCM patients were enrolled in a next-generation sequencing study that confirmed enrichment of truncating variants in *TTN* and identified additional pathogenic variants in *FLNC, DSP,* and *BAG3* (Goli et al., 2021). Moreover, limited family studies have identified pathogenic mutations in cardiomyopathy genes in families with both PPCM and DCM, supporting the concept that PPCM is a manifestation of familial DCM (van Spaendonck-Zwarts et al., 2014) that is caused by the extreme mechanical load placed on the pregnant heart. The overlap in genetic architecture, clinical features, and remodeling patterns support the classification of PPCM as a form of DCM (Ware et al., 2016; Seidman et al., 2025; Bollen et al., 2014).

**TABLE 1:** Sarcomere and cytoskeleton Genetic variants identified in PPCM patients.

Gene name	Variant type	Variant	DCM, PPCM, or both?	Citation
*BAG3*	Nonsense	Arg121*	PPCM	Goli et al. (2021)
*DES*	Nonsense	Arg429*	PPCM	Goli et al. (2021)
*DSP*	Frameshift	*Thr388fs*	PPCM	Goli et al. (2021)
*DSP*	Frameshift	*Glu1168GlyfsTer5*	PPCM	Goli et al. (2021)
*DSP*	Nonsense	Tyr249*	PPCM	Goli et al. (2021)
*DSP*	Nonsense	Trp 2056*	PPCM/DCM	Ware et al. (2016)
*FLNC*	Frameshift	*Ser1388fs*	PPCM	Goli et al. (2021)
*FLNC*	Frameshift	*Val2308fs*	PPCM	Goli et al. (2021)
*FLNC*	Nonsense	Arg1370*	PPCM	Goli et al. (2021)
*FLNC*	Splice site	*c.2015+1G>A*	PPCM	Goli et al. (2021)
*MYH6*	Frameshift	*Glu924fs*	PPCM	Goli et al. (2021)
*MYH6*	Nonsense	Arg855*	PPCM	Goli et al. (2021)
*MYH6*	Nonsense	Trp546*	PPCM/DCM	Ware et al. (2016)
*MYH7*	Frameshift	*Gly1006fs*	PPCM	Goli et al. (2021)
*MYH7*	Frameshift	Arg*1259fs*	PPCM	Goli et al. (2021)
*MYH7*	Nonsense	Glu1172*	PPCM/DCM	Ware et al. (2016)
*PSEN2*	Missense	Arg*62Cys*	PPCM/DCM	Morales et al., 2010)
*SCN5A*	Frameshift	*Asp 1816fs*	PPCM/DCM	Morales et al., 2010)
*SCN5A*	Frameshift	*Lys1578fs*	PPCM/DCM	Ware et al. (2016)
*SCN5A*	Nonsense	Glu904*	PPCM/DCM	Ware et al. (2016)
*TNNC1*	Missense	*Gln50Arg*	PPCM/DCM	van Spaendonck-Zwarts et al. (2014)
*TNNT2*	Frameshift	*Gln268fs*	PPCM/DCM	Ware et al. (2016)
*TNNT2*	Frameshift	Arg*92fs*	PPCM/DCM	Ware et al. (2016)
*TNNT2*	Missense	*Lys210del*	PPCM/DCM	Morales et al. (2010)
*TPM1*	Nonsense	Glu117*	PPCM/DCM	Ware et al. (2016)
*TTN*	Frameshift	*Trp18723fs*	PPCM/DCM	Ware et al. (2016)
*TTN*	Frameshift	Arg*20055fs*	PPCM	Goli et al. (2021)
*TTN*	Frameshift	*Lys29440fs*	PPCM	Goli et al. (2021)
*TTN*	Nonsense	Trp22234*	PPCM/DCM	Ware et al. (2016)
*TTN*	Nonsense	Arg5732*	PPCM/DCM	Ware et al. (2016)
*TTN*	Nonsense	Gln14475*	PPCM	Goli et al. (2021)
*TTN*	Nonsense	Gln25556*	PPCM	Goli et al. (2021)
*TTN*	Splice site	*c.63702+1G>T*	PPCM/DCM	Ware et al. (2016)
*TTN*	Splice site	*c.74014+2T>C*	PPCM/DCM	Ware et al. (2016)
*TTN*	Splice site	*c.87488-1G>A*	PPCM	Goli et al. (2021)
*VCL*	Frameshift	*Asn781fs*	PPCM	Goli et al. (2021)
*VCL*	Nonsense	Arg465*	PPCM/DCM	Ware et al. (2016)

Several genetically engineered mouse models have provided mechanistic insight into the transition from adaptive hypertrophy to PPCM, including cardiomyocyte-specific deletions of signal transducer and activator of transcription 3 (*Stat3*), peroxisome proliferator-activated receptor gamma coactivator 1-alpha (*Pgc-1α*), peptidyl tRNA Hydrolase (*Ptrh2*), coil helix coiled helix domain 10 (*Chchd10*), and heat shock protein 20 (*Hsp20)* (Table 2). (Montoya-Uribe et al., 2025; Sayles et al., 2026; Onusko et al., 2020; Hilfiker-Kleiner et al., 2007; Patten et al., 2012) The first experimental PPCM mouse model was a cardiomyocyte-specific *Stat3* deletion. *Stat3* is a transcription factor critical for cardiac survival, antioxidant defense, and ECM regulation (Hilfiker-Kleiner et al., 2007). In the postpartum heart, a loss of *Stat3* increases oxidative stress due to a reduction of manganese superoxide dismutase (MnSOD), a critical anti-oxidant enzyme localized in the mitochondrial matrix. MnSOD protects cells from reactive oxygen species (ROS) and the decreased levels promote an aberrant cathepsin D-mediated cleavage of prolactin leading to a 16-kDa vasoinhibin fragment with pro-apoptotic and anti-angiogenic properties (Hilfiker-Kleiner et al., 2007; Triebel et al., 2015). This fragment impairs VEGF and cardiac microvascular integrity leading to ventricular dysfunction to recapitulate a PPCM phenotype. Importantly, these findings provided the basis for therapeutic targeting of prolactin cleavage, as pharmacologic inhibition of prolactin release with Bromocriptine has been shown to prevent formation of the pathogenic 16-kDa fragment and improve cardiac function in experimental models (Hilfiker-Kleiner et al., 2012; Sliwa et al., 2010b) and is now currently undergoing a clinical trial (REBIRTH trial) (ClinicalTrials.gov, 2022).

**TABLE 2:** Genetically engineered mouse models of PPCM.

Model	Genetic alterations	Primary mechanism	Cardiac phenotype	Therapeutic implications	References
*Stat3* cKO	Cardiomyocytespecific *Stat3* deletion	↓ MnSOD/ROS ↑ Cathepsin D-mediated prolactin cleavage ↑ Vasoinhibin ↓ VEGF	↓ Microvascular density ↑ Cardiomyocyte apoptosis Capillary loss LV dysfunction	Bromocriptine inhibits prolactin cleavage ↓ Vasoinhibin production Improves cardiac function	Hilfiker-Kleiner et al. (2007)
*Pgc-1α* cKO	Cardiomyocytespecific *Pgc-1α* deletion	Impaired mitochondrial function and metabolism ↓ VEGF Defective angiogenesis	↑ Fibrosis ↑ Cardiomyocyte apoptosis Cardiac hypertrophy Systolic Dysfunction	VEGF injections + Bromocriptine reversed PPCM phenotype	Patten et al. (2012)
*Ptrh2* cKO	Cardiomyocytespecific *Ptrh2* deletion	↓ Integrin-mediated survival signaling ↓ PI3K-Akt ↓ Bcl-2	↑ Cardiomyocyte apoptosis ↑ Maladaptive Remodeling LV Dysfunction	Caspase-3 inhibitor reversed PPCM phenotype	Montoya-Uribe et al. (2025)
*Chchd10*	Heterozygous knock-in mutation p.S55 L	↓ Mitochondrial metabolite homeostasis Oxidative Phosphorylation Dysfunction	↑ Ventricular enlargement ↑ Fibrosis ↑ Myofibroblast differentiation	Dietary nicotinamide riboside + pterostilbene ↑ *Post partum* survival and cardiac energy metabolites	Sayles et al. (2026)
*Hsp20*	Transgenic *Hsp20* carrying S10 L mutation	↓ Βcl-2/Bax ↓ Akt Signaling	↑ Cardiomyocyte apoptosis Cardiac hypertrophy LV Dysfunction	Probenecid treatment reversed PPCM phenotype	Onusko et al. (2020), Liu et al. (2018)

Angiogenesis in cardiac tissue is also dysregulated in PPCM. One regulator of angiogenesis is peroxisome proliferator-activated receptor gamma coactivator 1-alpha (PGC-1α). PGC-1α is highly expressed in the heart and is responsible for inducing the VEGF expression and secretion allowing for the development of blood vessels that are necessary for mother and developing fetus (Chinsomboon et al., 2009; Arany et al., 2008; Arany et al., 2005). Maternal mice lacking cardiac *Pgc-1α* develop hypertrophic and fibrotic hearts with systolic dysfunction, indicative of PPCM (Patten et al., 2012). Treatment with VEGF injections and Bromocriptine reverse the PPCM phenotype in *Pgc-1α* cardiac KO mice.

The maternal heart is also protected by mitochondrial metabolic adaptation and cardiomyocyte stress-adaptive pathways. A recent heterozygous knock-in mouse model carrying the pathogenic p. S55 L mutation in the *Chchd10* (D10^S55L^) (Sayles et al., 2026) gene developed postpartum cardiac dysfunction adverse remodeling, energetic insufficiency marked by depletion of NAD(H), ADP, and heme levels, and increased maternal mortality. Coiled-coil-helix-coiled-coil-helix domain containing 10 (Chdchd10) is a mitochondrial protein required for maintaining mitochondrial architecture and energy production (Genin et al., 2016), making it critical for meeting the increased metabolic demands imposed on the maternal heart during pregnancy. Similarly, studies using *Hsp20*-S10 F mutant mice further demonstrated that pregnancy unmasks latent defects in cardiomyocyte stress-adaptive pathways. Heat shock protein 20 (Hsp20), a small stress-response chaperone that protects cardiomyocytes against oxidative stress (Fan et al., 2008), apoptosis (Fan et al., 2004), and pathological remodeling (Fan et al., 2006), was disrupted by the human *HSP20*-S10 F mutation and lead to a PPCM phenotype, such as LV dilation, hypertrophic remodeling, reduced ejection fraction and fractional shortening, and experienced severe postpartum mortality (Liu et al., 2018). Together, these findings point to dysregulated stress responses and metabolic cues in the transition from adaptive to pathological remodeling triggering PPCM.

A recent report in *Nature Communications* identified *Ptrh2* as a regulator of cardiac function that when deleted specifically in the heart recapitulates PPCM in maternal mice (Montoya-Uribe et al., 2025). *PTRH2* was also downregulated at mRNA and protein levels in several PPCM patient hearts compared to healthy control hearts. In the mouse model loss of *Ptrh2* leads to disruption of integrin-associated pro-survival signaling leads thereby activating maladaptive stress responses (Montoya-Uribe et al., 2025). Significantly, *Ptrh2* expression is dynamically regulated during pregnancy. In wild-type female mice, cardiac *Ptrh2* mRNA and protein levels significantly increase during late gestation, coinciding with peak hemodynamic load, and both mRNA and protein return to pre-pregnancy levels in the postpartum period. In the pregnant heart, this dynamic regulation of Ptrh2 expression points to a role in responding to cardiac mechanical stress to regulate the healthy adaptive program in the maternal heart. In a cardiomyocyte-specific *Ptrh2* knock-out mouse model, all maternal females develop progressive cardiac pathology that recapitulates human PPCM. Indeed, loss of *Ptrh2* in the heart shifts the trajectory from adaptive to maladaptive remodeling under physiological volume overload in the maternal heart. These data suggest that PTRH2 plays a cardioprotective role during pregnancy, and its loss abolishes this safeguard impairing the heart’s ability to adapt to the exacerbated mechanical stresses of pregnancy.

Importantly, based on these pre-clinical models, *STAT3, PGC-1α, PTRH2, CHCHD10,* and *HSP20* may be dysregulated in PPCM patients; however, large-scale genetic, WES and translational studies are needed to determine whether mutations or regulatory defects in these genes directly contribute to PPCM. Future work focusing on the identification and validation of molecular markers that govern the transition from adaptive to maladaptive remodeling will be essential for the development of targeted, mechanism-based therapies for PPCM.

## Discussion

PPCM arises when the maternal heart fails to adapt to the extreme hemodynamic and mechanical stresses of late pregnancy and the early postpartum period. Physiological cardiac hypertrophy during pregnancy is a finely tuned adaptive response regulated, in part, by integrin-mediated mechanotransduction and downstream pro-survival signaling. Together, these mechanisms preserve cardiomyocyte survival, maintain contractile function, and allow reversible remodeling. Dysregulation, through genetic mutations, oxidative stress, or impaired signaling shifts hypertrophy from adaptive to maladaptive, leading to DCM, fibrosis, reduced contractile function and HF.

A key factor in PPCM progression is cardiomyocyte apoptosis, which is mediated in part, by caspase activation. The observed increased cardiomyocyte cell death is supported by pre-clinical mouse models (Hilfiker-Kleiner et al., 2007; Patten et al., 2012; Hilfiker-Kleiner et al., 2012; Arany et al., 2008; Russell et al., 2004). To further investigate the role of apoptosis in pregnancy-associated cardiac dysfunction, pregnant mice with cardiac-specific overexpression of Gaq (Biliran et al., 2008) were treated with a polycaspase inhibitor (IDN-1965), on day 12 of pregnancy and continued treatment until postpartum day 14 (Jan et al., 2004). Although this model does not fully recapitulate human PPCM and is not fully being further investigated (Elkayam, 2011), treatment with a polycaspase inhibitor suppressed the activity of all caspase enzymes, reduced cardiomyocyte apoptosis, and improved cardiac function in this mouse model.

Evidence for the importance of apoptotic regulation in PPCM is further supported by the *Hsp20*-S10 F mutant mouse model, where disruption of a key cardioprotective chaperone increased susceptibility to pregnancy-induced cardiac stress (Liu et al., 2018). Rather than maintaining normal pro-survival signaling, the mutant heart exhibited enhanced apoptotic remodeling, reflected by reduced Bcl-2/Bax ratios and progressive postpartum cardiac dysfunction. A follow-up study using the *Hsp20*-S10F postpartum mouse model found maladaptive calcium handling may contribute to PPCM progression. Postartum *Hsp20*-S10F maternal mice had increased expression of the transient receptor potential vanilloid 2 (TRPV2), a stretch and agonist-activated calcium channel (Onusko et al., 2020; Caterina et al., 1999). Pharmacological intervention with probenecid, partially rescued these effects, improving survival by reducing apoptosis and pathological remodeling. Probenecid is an agonist that stimulates TRVP2 and causes increased cytosolic calcium (Koch et al., 2012). Recent work using the *Chchd10-S55L* mitochondrial cardiomyopathy mouse model found that supplementation with nicotinamide riboside and pterostilbene partially rescued postpartum survival and improved cardiac metabolic homeostasis by restoring mitochondrial energetic balance (Sayles et al., 2026). Together, these studies highlight mitochondrial metabolism as promising therapeutic targets for mitigating maladaptive remodeling and cardiac dysfunction in PPCM.

**FIGURE 4:**
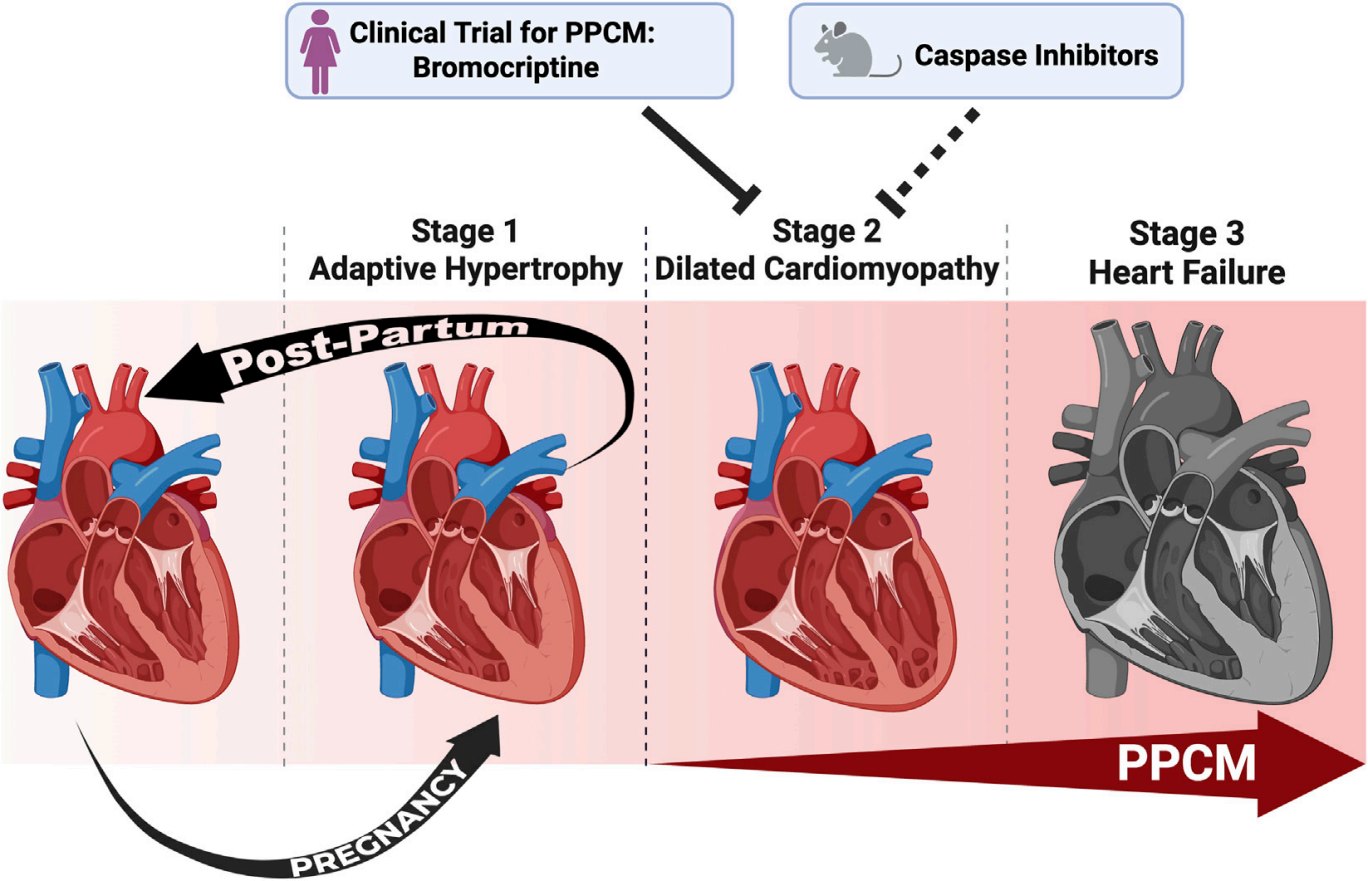
PPCM Progression from Adaptive Remodeling to Heart Failure, Highlighting Therapeutic Interventions. Pregnancy-induced hemodynamic stress normally promotes a reversible state of adaptive hypertrophy (Stage 1), allowing the maternal heart to accommodate increased circulatory demand and return to baseline in the postpartum period. In PPCM, this adaptive response is destabilized, leading to DCM (Stage 2) and HF (Stage 3). This transition is driven by impaired mechanotransduction and pro-survival signaling, resulting in increased cardiomyocyte apoptosis. A current clinical trial targets prolactin signaling with Bromocriptine. Pre-clinical mouse models of PPCM were rescued by caspase three inhibition. Created in BioRender. Matter, M (2026) https://BioRender.com/i424prf.

In the *Ptrh2* cardiac specific mouse KO model pharmacological inhibition specifically of caspase-3 significantly rescued the PPCM phenotype in postpartum *Ptrh2*-CKO mice (Montoya-Uribe et al., 2025). Continuing treatment with a caspase-3 specific inhibitor (Z-DEVD-FMK) on day 10 of pregnancy and continuing until birth significantly reduced cardiomyocyte apoptosis, limited fibrotic remodeling, and improved systolic function. Indeed, the maternal mice treated with the caspase three specific inhibitor survived the postpartum period whereas all *Ptrh2*-CKO maternal mice treated with the inactive control peptide (Z-FA-FMK) died postpartum. Taken together, these findings confirm a significant role for caspase-mediated cell death in PPCM disease progression (Hayakawa et al., 2003) (Figure 4).

Clinical trials for new PPCM therapies are minimal, and guideline-directed HF therapy remains central to PPCM treatment. The first clinical trial for PPCM is ongoing. It is a randomized evaluation of Bromocriptine in myocardial recovery therapy (the REBIRTH trial) (Pearson et al., 2000) (Figure 4). This trial will evaluate Bromocriptine’s effects and clinical outcomes in PPCM. Bromocriptine blocks prolactin-mediated pathways to inhibit the aberrant cleavage of prolactin into the 16-kDa vasoinhibin fragment. This randomized, placebo-controlled PPCM clinical trial will report its findings on whether it improved myocardial recovery and clinical outcomes in women with newly diagnosed PPCM in 2029.

Beyond PPCM, genes dysregulated in the maternal heart may have a broader role in the development of HF under other conditions of chronic mechanical or volume overload, such as hypertensive heart disease, valvular dysfunction, or post-infarction remodeling. Given that integrin-dependent mechanotransduction and pro-survival signaling are critical determinants of cardiomyocyte resilience, loss or dysregulation of genes involved in these pathways may contribute to maladaptive remodeling in these contexts as well. Moreover, the role of mechano-metabolic coupling, mitochondrial calcium handling, and regulation of cardiac energy homeostasis during the heightened physiological demands of pregnancy are being intensely investigated. Further studies into how these pathways coordinate stress adaptation, metabolic remodeling, and mitochondrial function in the maternal heart will be critical for understanding why some individuals progress to PPCM and heart failure while others are able to recover. In addition, large-scale genomic and transcriptomic screening in PPCM patient populations may help uncover disease-associated pathways, genetic susceptibilities, and molecular drivers of cardiac dysfunction all of which may lead to effective therapeutic targets. Collectively, these findings underscore the importance of mechanotransduction and cardiomyocyte stress-adaptive pathways in maintaining maternal cardiac resilience during pregnancy. Due to overlap between PPCM symptoms and those commonly experienced during healthy pregnancy, early diagnosis remains challenging. Future efforts focused on identifying PPCM-specific biomarkers from large scale genomic screenings may also improve early clinical detection, risk stratification, and intervention. Advancing these areas of investigation will be essential for developing specific therapies to block the progression to HF from PPCM or other stresses that trigger HF due to high hemodynamic load (pressure and volume overload) including Left Ventricular Hypertrophy (LVH), Dilated Cardiomyopathy (DCM) and Hypertensive Heart Disease (HHD).
